# Macrophage metabolism in the intestine is compartment specific and regulated by the microbiota

**DOI:** 10.1111/imm.13461

**Published:** 2022-03-11

**Authors:** Nicholas A. Scott, Melissa A. E. Lawson, Ryan James Hodgetts, Gwénaëlle Le Gall, Lindsay J. Hall, Elizabeth R. Mann

**Affiliations:** ^1^ Division of Infection, Immunity and Respiratory Medicine Faculty of Biology, Medicine and Health School of Biological Sciences University of Manchester Manchester Academic Health Science Centre Lydia Becker Institute of Immunology and Inflammation Manchester UK; ^2^ Gut Microbes & Health Quadram Institute Bioscience Norwich Research Park Norwich UK; ^3^ Department of Immunology Weizmann Institute of Science Rehovot Israel; ^4^ Norwich Medical School University of East Anglia Norwich Research Park Norwich UK; ^5^ ZIEL – Institute for Food & Health Technical University of Munich Freising Germany

**Keywords:** gut, macrophage, metabolism, microbiota

## Abstract

Intestinal macrophages play a vital role in the maintenance of gut homeostasis through signals derived from the microbiota. We previously demonstrated that microbial‐derived metabolites can shape the metabolic functions of macrophages. Here, we show that antibiotic‐induced disruption of the intestinal microbiota dramatically alters both the local metabolite environment and the metabolic functions of macrophages in the colon. Broad‐spectrum antibiotic administration in mice increased the expression of the large neutral amino acid transporter LAT1 and accordingly, amino acid uptake. Subsequently, antibiotic administration enhanced the metabolic functions of colonic macrophages, increasing phosphorylation of components of mammalian/mechanistic target of rapamycin signalling pathways, with increased expression of genes involved in glycolysis and oxidative phosphorylation (OXPHOS), increased mitochondrial function, increased rate of extracellular acidification (ECAR; measure of glycolysis) and increased rate of oxygen consumption (OCR; measure of OXPHOS). Small bowel macrophages were less metabolically active than their colonic counterparts, with macrophage metabolism in the small intestine being independent of the microbiota. Finally, we reveal tissue‐resident Tim4^+^ CD4^+^ macrophages exhibit enhanced fatty acid uptake alongside reduced fatty acid synthesis compared to recruited macrophages. Thus, the microbiota shapes gut macrophage metabolism in a compartment‐specific manner, with important implications for monocyte recruitment and macrophage differentiation.

## Graphical abstract

Antibiotic‐induced disruption of the intestinal microbiota dramatically alters the local metabolite environment in the intestine and metabolic functions of intestinal macrophages. These effects are compartment specific, with a profound effect on colonic macrophages rather than their small intestinal counterparts. The capacity of antibiotics to modulate intestinal macrophage metabolic function was also subset specific, limited to recruited versus resident macrophages.

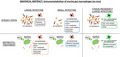

## INTRODUCTION

Intestinal macrophages in the lamina propria are specialized to remain hyporesponsive to the gut microbiota, while remaining poised to respond to pathogens when required. Under steady‐state conditions, intestinal macrophages do not respond to bacterial stimulation, such as lipopolysaccharide [1, 2], thought to be one of the major mechanisms by which harmful immune responses against the intestinal microbiota are avoided. Most macrophages within the intestine are seeded from Ly6C^+^MHCcII^−^ blood circulating monocytes, which differentiate to an intermediate stage (Ly6C^+^MHCcII^+^), before maturing to macrophages (Ly6C^−^MHCcII^+^) [3]. However, Tim4 and CD4 expression identifies macrophages with low turnover from the circulation [4], although the proportions of these macrophages differ between the small and large intestines and functional differences between recently recruited and long‐lived macrophages in the intestine are unclear.

Metabolic pathways provide energy to cells but are also capable of regulating immune function. Immune cell metabolism regulates their function and is dependent on the local availability of nutrients [5, 6, 7, 8]. Classically activated macrophages are typically induced by lipopolysaccharide (LPS) or interferon gamma (IFNγ) [9], defined by the production of high levels of pro‐inflammatory cytokines in response to pathogens, rely on glucose utilization for energy (anaerobic glycolysis). In contrast, alternatively activated macrophages provide growth factors for wound healing and are generated by type 2 cytokines such as interleukin (IL)‐4 or IL‐13, using oxidative phosphorylation (OXPHOS) generated within mitochondria from the tricarboxylic acid cycle (TCA) as their primary energy source [10, 11]. Primary tissue macrophages exhibit properties of both classical and alternative activation, and it is now thought that a spectrum of macrophage phenotypes exist which are shaped by local tissue niches.

Amino acids, building blocks of proteins and nucleotides, govern cellular metabolism by forming precursors of key regulatory metabolites [12]. Amino acid uptake has been proposed to be important for macrophage function, as LPS stimulation of bone marrow‐derived macrophages increases the expression of Slc7a5, a component of the System L‐type large neutral amino acid transporter LAT1, and subsequently, leucine importation through LAT1 regulates pro‐inflammatory cytokine production by macrophages [13]. This is of particular relevance to the intestine with high levels of amino acids from the intake of dietary protein. It was recently demonstrated that deletion of Slc3a2, a subunit of amino acid transporters including LAT1, in CX3CR1^+^ gut macrophages reduces their numbers and impedes monocyte differentiation to mature MHC cII^+^ macrophages [14].

The gut microbiota generates metabolites that regulate the intestinal immune system, with arguably the most extensively studied being short‐chain fatty acids (SCFAs) [15, 16, 17, 18]. We and others have demonstrated the SCFA butyrate induces metabolic rewiring in intestinal macrophages towards OXPHOS [19] and away from glycolysis [20]. However, the overall contribution of the microbiota to macrophage metabolism in the intestine remains unclear. Here, we demonstrate that antibiotic‐induced disruption of the intestinal microbiota enhances amino acid uptake by macrophages and their metabolic capacity, with upregulation of glycolysis as well as OXPHOS pathways. These changes were not SCFA dependent and were limited to colonic macrophages only. Indeed, the metabolic capacity of macrophages in the small intestine was diminished compared to that of macrophages in the colon. The importance of considering macrophage subsets in the intestine is demonstrated with fatty acid uptake being a defining characteristic of long‐lived Tim4^+^CD4^+^ colonic tissue‐resident macrophages.

## METHODS

### Mice

WT C57BL/6 mice (Charles River) were maintained under specific pathogen‐free conditions at the University of Manchester, UK. Age‐ and gender‐matched adult (8–12 weeks old, female) animals were used in all experiments and protocols approved by the University of Manchester Animal Welfare Ethical Review Boards. All experiments were carried out under license issued by and according to UK Home Office regulations. Sample sizes were determined based on previous experiments investigating the effects of antibiotics on intestinal macrophage function, to detect a 20% difference with ~10% standard deviation.

### Antibiotic treatment

Mice were treated with an antibiotic cocktail containing ampicillin (1 g/L), metronidazole (1 g/L), neomycin (1 g/L), gentamicin (1 g/L) and vancomycin (0.5 g/L) in drinking water for 7 days, which was replenished once on day 3 or 4. Control (untreated) mice were used from the same batch of mice bought from Charles River as those that were antibiotic treated and housed in the same location (within separate cages as antibiotics administered in drinking water).

### Isolation of intestinal lamina propria cells

Large and small intestines were removed from mice, cleaned with phosphate‐buffered saline (PBS) and chopped into 5‐mm sections before incubation with 2 mm ethylenediaminetetraaceticacid in HBSS three times for 15 min on shake at 210 rpm. After washing, tissue was digested with 1·25 mg/ml collagenase D (Sigma), 0·85 mg/ml collagenase V (Sigma) and 1 mg/ml dispase (Gibco, Invitrogen) in 10% fetal bovine serum (FBS) RPMI‐1640 (Sigma) at 37°C for 30 min (small intestine) or 35 min (large intestine).

### Faecal metabolite analysis

Faecal metabolite analysis was performed as described previously [19]. Briefly, stools were prepared for ^1^H nuclear magnetic resonance (NMR) spectroscopy and recorded at 600 MHz on a Bruker Avance spectrometer (Bruker BioSpin GmbH) running Topsin 2.0 software and fitted with a broadband inverse probe. All metabolites were quantified using Chenomx NMR suite 7.6 software and using the 2D‐NMR methods, COSY, HSQC and HMBC.

### Flow cytometry and sorting of cells

Isolated cells were stained with Fc block (BD Sciences) for 5 min prior to staining at 4°C in the dark using the antibodies listed in Table S1 and analysed using an LSR Fortessa cytometer (BD Biosciences) and FlowJo software (BD). Monocytes and macrophages were sorted using a FACS Aria Fusion as live gated CD45^+^SiglecF^−^Ly6G^−^CD11b^+^CD64^+^ cells to >97% purity and further subdivided into subsets based on MHC class II, Ly6C, Tim4 and CD4 expression.

### SCFA and amino acid treatment

Mice were administered acetate (67·5 mm), propionate (25·9 mm) and butyrate (40 mm) in drinking water (concentrations reflective of ratios within the intestine [21]) with or without antibiotics for 7 days. For the amino acid supplementation experiments, mice received 100 mm each of lysine, leucine and glutamine or isoleucine, tryptophan (30 mm) and glutamine in drinking water with or without antibiotics for 7 days.

### Amino acid uptake assay

System L amino acid transport was measured using the fluorescent properties of kynurenine.^22^ After staining to distinguish macrophages, each sample was split into four tubes: HBSS alone (FMO/background), l‐kynurenine (Sigma; 200 μm final), l‐kynurenine + leucine (Sigma, 5 mm final) and kynurenine + 2‐amino‐2‐norbornanecarboxylic acid (BCH)—a LAT1 inhibitor (Sigma; 10 mm final). Samples were placed at 37°C for 5 min, before addition of Cytofix Fixation Buffer (BD Biosciences), pulse vortex and incubation at 4°C for 20 min. After washing, cells were analysed by flow cytometry. Kynurenine was detected on the e450/BV421 channel (BV450/50 filter on LSR Fortessa).

### Phosphoflow

Cell suspensions were lysed, fixed and stained using a modified BD Phosphoflow^TM^ protocol. After addition of pre‐warmed lyse/fix buffer (BD Phosphoflow^TM^) cells were inverted 10 times and incubated at 37°C for 10 min. Cells were washed and centrifuged 600 ***g*** for 6 min twice before permeabilizing with Perm Buffer III for 30 min at 4°C. After washing, cells were treated with Fc block for 5 min before staining with Phosphoflow^TM^ and regular antibodies (see antibody list, Table S1).

### Cellular metabolism assays

Intestinal cell suspensions were stained with an antibody cocktail to distinguish macrophages before the addition of Mitotracker Green (50 nm final) and tetramethylrhodamine, ethyl ester (TMRE, 25 nm final) in 10% FBS RPMI‐1640 and incubation for 30 min at 37°C with CO_2_. Cells were washed twice before analysis by flow cytometry. Mitotracker Green was detected on the FITC channel (B530/30 filter on LSR Fortessa) and TMRE on the PE channel (Y568/15 filter on LSR Fortessa). For fatty acid uptake, 5 μm BODIPY FL C16 (Invitrogen) in 0·5% fatty acid‐free BSA RPMI‐1640 (both Sigma) was added to cells for 10 min in a 37°C 5% CO_2_ incubator and signal detected on the FITC channel (B530/30 filter on the LSR Fortessa). For glucose uptake, 100 μm 2‐NBDG was added to cells in glucose‐free RPMI‐1640 media (Gibco, ThermoFisher Scientific) for 1 h at 37°C in an incubator and detected on the FITC channel (B530/30 filteron the LSR Fortessa).

### Quantitation of gene expression by real‐time PCR

Total RNA was extracted from monocytes and macrophages from the intestines of individual or pooled samples using the RNeasy Micro Kit (Qiagen). RNA were reverse transcribed to cDNA using the High Capacity RNA‐to‐cDNA Kit (Applied Biosystems, Thermo Scientific) and gene expression was assayed using quantitative reverse transcription PCR using PerfeCTa SYBR Green Fastmix Low ROX (Quanta) on the QuantStudio 12K Flex (Applied Biosystems) with primers (Integrated DNA Technologies) listed in Table S2.

### Phagocytosis assay

After digestion, intestinal cell suspensions were placed in 10% FBS RPMI‐1640 in a 5% CO_2_ 37°C incubator to allow time for attachment to the tubes for 1·5–2 h. After washing, 100 μl of 1 mg/ml pHrodo Red *Escherichia coli* BioParticles (Invitrogen) were added in PBS and cells placed at 4°C (negative control) or 37°C for 30 min. After washing, cells were stained with the macrophage antibody panel and analysed by flow cytometry. pHrodo Red BioParticles were detected on the PE channel (Y568/15 filter, LSR Fortessa).

### Seahorse

Extracellular acidification rates (ECAR) and oxygen consumption rates (OCR) were measured using an XF‐96 Extracellular Flux Analyser (Seahorse Bioscience, Agilent Tecnologies). ECAR was measured at baseline and after addition of 25 mm glucose, 1–2 μm oligomycin and 50 mm 2‐deoxy‐d‐glucose). OCR were taken under basal conditions and following the addition of 1–2 μm oligomycin, 1.5 μm fluro‐carbonyl cyanide phenylhydrazone, 200 μm etomoxir and 100 nm rotenone + 1 μm antimycin A (all purchased from Sigma). Each Seahorse experiment contains ex vivo pooled and sorted live CD45^+^SiglecF^−^Ly6G^−^CD11b^+^CD64^+^ intestinal cells isolated from five or six mice per group.

## RESULTS

### Antibiotic treatment increases amino acid uptake and expression of the LAT1 transporter on colonic macrophages

**FIGURE 1:**
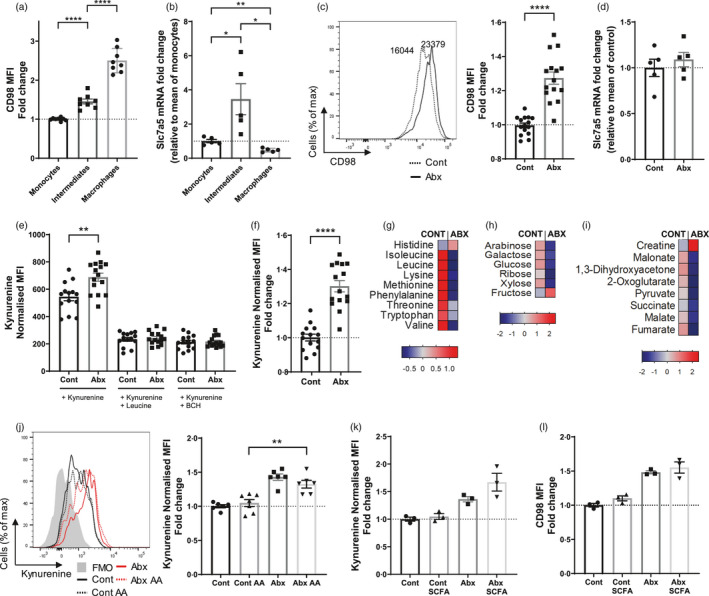
Antibiotic treatment alters amino acid transport in large intestinal macrophages. (a–f) Mice were treated with antibiotic or control water and colonic monocyte‐macrophage populations (gated from live CD45^+^Ly6G^−^CD11b^+^CD64^+^) characterized by flow cytometry or sorted for qPCR. (a) Mean fluorescent intensity (MFI) of CD98 protein expression at steady state on monocytes (Ly6C^+^MHC cII^−^), intermediates (Ly6C^+^MHC cII^+^) and macrophages (Ly6C^−^MHC cII^+^). Fold change is relative to mean monocyte readings; *n* = 8. (b) Slc7a5 mRNA expression on same subsets shown in (a). Fold change is relative to mean monocyte readings; *n* = 5. (c) Mean fluorescent intensity of CD98 protein expression on macrophages (Ly6C^−^MHC cII^+^). Fold change is relative to mean control readings; *n* = 15. (d) Slc7a5 mRNA expression on Ly6C^−^MHC cII^+^ macrophages. Fold change is relative to mean of control group; *n* = 5. (e) Intestinal cell suspensions were treated with 200 µm kynurenine and cell uptake measured on Ly6C^−^MHC cII^+^ intestinal macrophages at 450 nm in the presence or absence of competitive leucine (5 mm) or the System L transport inhibitor BCH (10 mm). MFI readings were normalized to no kynurenine samples; *n* = 15. (f) Data from (e) (kynurenine alone) expressed as fold change relative to control treated, *n* = 15. (g–i) Mice were treated with antibiotic or control water and faecal pellets analysed by nuclear magnetic resonance (NMR) spectroscopy. Pooled data depicting heat maps made from *Z*‐scores (relative). (g) Fold change in selected amino acids. (h) Fold change in selected sugars. (i) Fold change in selected energy metabolites. (j–l) In addition to control and antibiotic water alone, separate cohorts of mice were treated with control and antibiotic water supplemented with amino acids (AA) (leucine, lysine and glutamine) (j) (*n* = 6–7), or short‐chain fatty acids (SCFA) comprising butyrate (40 mm), acetate (67.5 mm) and propionate (25 mm) (k) (*n* = 3). MFI readings are normalized to no kynurenine samples and fold change expressed as mean of control samples. (l) MFI of CD98 protein expression on colonic macrophages from (k). Fold change is normalized to mean control readings; *n* = 3. *p < 0·05, **p < 0·01, ****p < 0·0001, Student's *t*‐test

Given the high levels of amino acids in the intestine and contribution of components of the System L amino acid transporter LAT1 to the differentiation of intestinal macrophages [14], we first examined amino acid transport in monocytes and macrophages of the intestine. Leucine uptake promotes pro‐inflammatory cytokine production in macrophages [13], with importation of leucine into cells occurring via the transporter LAT1, a heterodimer of CD98 and Slc7a5. We assessed the expression of CD98 and Slc7a5 in the CD11b^+^CD64^+^ monocyte and macrophage subsets from the colon (for gating strategy, see Figure S1). Protein and transcript levels of CD98 and Slc7a5, respectively, increased significantly as monocytes (Ly6C^+^MHCcII^−^) matured to intermediate monocytes (Ly6C^+^MHCcII^+^) and increased further for CD98 following macrophage differentiation (Ly6C^−^MHCcII^+^) (Figure 1a,b), inferring amino acid transport may be enhanced during maturation of monocytes.

To assess whether components of the LAT1 amino acid transport pathway may be modulated by the microbiota, we disrupted the gut microbiota by treating mice with a broad‐spectrum antibiotic cocktail (ampicillin, metronidazole, gentamicin, vancomycin and neomycin) for 7 days. CD98 was upregulated on macrophages from antibiotic‐treated mice compared to controls (Figure 1c), but Slc7a5 expression was not significantly changed (Figure 1d). The uptake of amino acids was quantified by measuring the importation of kynurenine, a florescent compound selectively transported by LAT1 [22], assessed by flow cytometry in CD11b^+^CD64^+^MHC cII^+^Ly6C^−^ colonic macrophages. Macrophages from antibiotic‐treated mice demonstrated increased kynurenine (LAT1‐mediated amino acid) uptake (Figure 1e,f) which the addition of competing leucine or the System L inhibitor, 2‐amino‐2‐norbornanecarboxylic acid (BCH), blocked (Figure 1e).

To assess whether enhanced uptake of amino acids following antibiotic treatment may be a result of changes in the concentration of intestinal amino acids, we assessed the local levels of amino acids and energy‐related products by analysing faecal pellets using NMR spectroscopy. Antibiotic treatment depleted the levels of amino acids in the colon (Figure 1g) including leucine which is important for the production of pro‐inflammatory cytokines by monocytes and macrophages [13]. In addition, glucose, galactose and ribose were all reduced, among other sugars (Figure 1h), and several other key metabolites declined, such as succinate, generated during mitochondrial respiration in the TCA, and pyruvate, generated by glycolysis. Interestingly, one of the few molecules elevated is creatine, a catabolite of arginine recently shown to promote M (IL‐4) polarization [23]. Collectively these data demonstrate that disruption of the microbiota in the gut drastically changes the availability of local metabolites that have been shown to modulate macrophage function.

To investigate whether supplementation of amino acids in drinking water with antibiotics may restore the normal levels of amino acid uptake, mice were administered antibiotic or control water supplemented with System L amino acids (leucine, glutamine and lysine). Within both the control and antibiotic groups, the addition of amino acids had no effect on kynurenine (amino acid) uptake in macrophages (Figure 1j), suggesting that administration of these amino acids in vivo does not alter amino acid uptake by colonic macrophages. However, supplementation of drinking water with the amino acids leucine, glutamine and lysine (combination 1) in naïve mice reduced mitochondrial mass (Mitotracker Green assay). Supplementation of other LAT1 transported amino acids (isoleucine, glutamine and tryptophan; combination 2) did not alter mitochondrial mass (Figure S2a) but did reduce fatty acid uptake (BODIPY uptake assay; Figure S2c). Thus, different amino acids have the capacity to shape different aspects of colonic macrophage metabolism.

The concentrations of SCFAs including butyrate, propionate and acetate are also decreased with the same antibiotic regimen, with SCFAs having potent effects on macrophage function [19]. To investigate whether changes in local SCFA levels regulate amino acid transport, SCFAs, namely acetate, butyrate and propionate were administered to antibiotic‐treated and control mice to examine their effect on amino acid uptake and CD98 expression. The addition of SCFA did not change kynurenine transport (Figure 1k) or CD98 protein levels (Figure 1l) in colonic macrophages. However, the supplementation of SCFAs in the drinking water of naïve mice altered macrophage metabolism, reducing mitochondrial mass and potential (TMRE assay), as well as reducing fatty acid uptake (BODIPY uptake assay; Figure S2), highlighting the ability of microbial metabolites to regulate intestinal macrophage metabolism.

These data demonstrate antibiotic‐induced disruption of the microbiota enhances amino acid uptake in colonic macrophages. While amino acid and SCFA supplementation did not alter macrophage uptake of amino acids, these molecules regulated mitochondrial mass and function alongside fatty acid uptake, suggesting that antibiotic‐induced alterations in local amino acid and SCFA levels may impact the metabolic capacity of macrophages in the intestine.

### Antibiotics enhance the metabolic capacity of colonic macrophages

**FIGURE 2:**
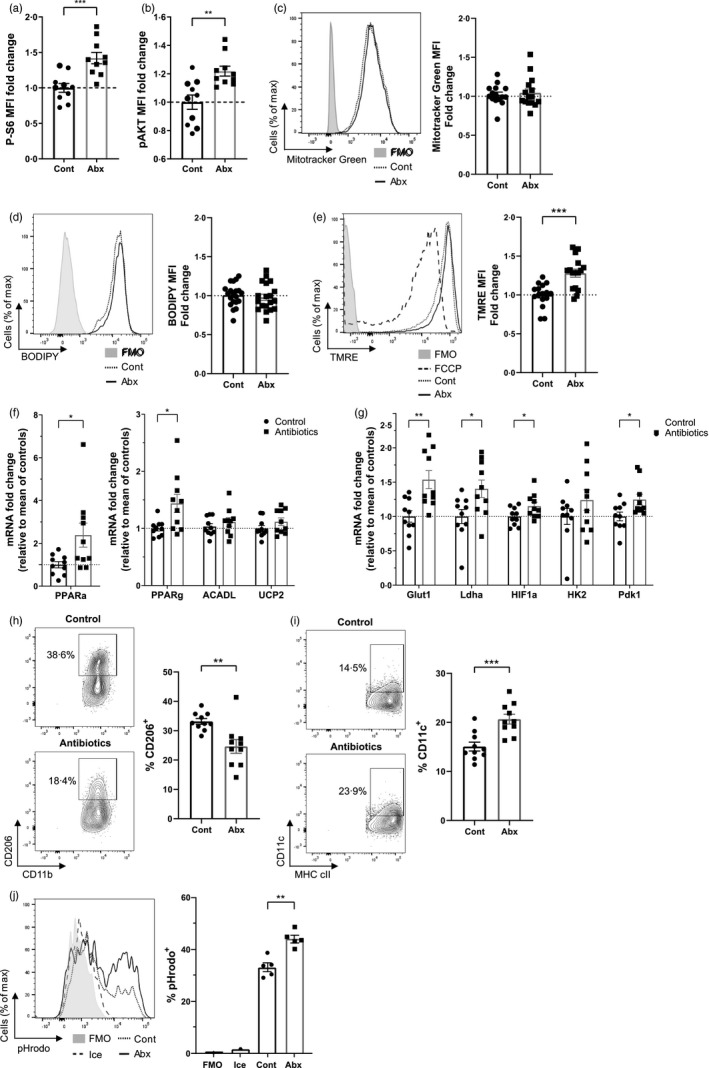
Antibiotics alter the metabolism *o*f macrophages within the large intestine. Mice were treated with antibiotic or control water and large intestinal cell suspensions isolated. (a–e) Macrophages (live CD45^+^SiglecF^−^Ly6G^−^CD11b^+^CD64^+^Ly6C^−^MHC cII^+^) were identified by flow cytometry for gating. (a, b) Phosphoflow for phosphorylated serine‐6‐kinase (a) and phosphorylated AKT (b). Fold change is normalized to control samples; *n* = 10. (c) Cells were treated with 50 nm Mitotracker Green and staining measured at 530 nm. *n* = 10. (d) Cells were treated with 5 µm BODIPY FL C16 in fatty acid‐free media, and uptake measured at 530 nm, *n* = 19–20. (e) Cells were treated with 25 nm of TMRE and fluorescence measured at 586 nm. *n* = 16. (f, g) Large intestinal monocyte‐macrophages (live CD45^+^SiglecF^−^Ly6G^−^CD11b^+^CD64^+^) were sorted by flow cytometry, and mRNA expression of glycolytic‐related (f) and oxidative phosphorylation‐related (g) genes was quantified by real‐time PCR. Fold change is relative to mean of control samples; *n* = 10. (h, i) CD206 (h) and CD11c (i) expression and on Ly6C^−^MHCcII^+^ macrophages by flow cytometry; *n* = 10. (j) Cell suspensions were analysed with the pHrodo phagocytosis assay and uptake on macrophages measured at 561 nm; *n* = 5.*p < 0·05, **p < 0·01, ***p < 0·001, Student's *t*‐test

The modulation of cellular metabolism is mediated through mechanistic/mammalian target of rapamycin (mTOR), with amino acids (in particular leucine) enhancing the activity of mTOR complex 1 (mTORC1) through the acetylation of raptor by its metabolite acetyl‐coenzyme A [24]. This complex functions as a key nutrient‐sensing pathway that permits macrophages to regulate cell growth and metabolism. To investigate whether increased System L transported amino acid uptake in intestinal macrophages results in increased mTORC1 activity, we analysed the phosphorylation of S6‐kinase (S6), which is phosphorylated downstream of mTORC1. Colonic macrophages isolated from antibiotic‐treated mice had higher levels of phosphorylated‐S6 than macrophages from control animals (Figure 2a). mTORC1 is also activated by growth factors and cytokine signalling, through the PIP3K‐Akt (class I phosphoinositide 3‐kinase‐Akt) pathway upstream of mTORC1. Accordingly, we also revealed increased phosphorylation of Akt in gut macrophages from antibiotic‐treated mice (Figure 2b).

Following antibiotic treatment, features of glycolysis and OXPHOS were measured using a range of experimental cellular assays. Although there was no difference in mitochondrial mass and fatty acid uptake (Figure 2c,d), antibiotics significantly enhanced the mitochondrial function of colonic macrophages (Figure 2e), suggestive of increased levels of OXPHOS. The gene expression of OXPHOS regulatory genes were evaluated by qPCR in CD11b^+^ CD64^+^ colonic monocyte‐macrophages. Antibiotic‐treated mice had elevated expression of two enzymes involved in transcriptional regulation of fatty acid oxidation (*Ppara* and *Ppary*) (Figure 2f), and heightened expression of key glycolysis genes, including *Glut1* (primary glucose transporter), *Ldha* (lactate dehydrogenase—catalyses the final step of glycolysis), *Hif1a* (hypoxia‐inducible factor 1‐alpha—a key transcription factor that targets glucose uptake and glycolysis) and *Pdk1* (pyruvate dehydrogenase kinase 1—inhibits TCA cycle; Figure 2g). Despite OXPHOS being a characteristic of alternatively activated macrophages [25], the mannose receptor (CD206), a marker of alternative activation, was downregulated in macrophages from antibiotic‐treated mice (Figure 2h) and CD11c, a marker of classical activation, was significantly increased (Figure 2i). However, corresponding with enhanced characteristics of classical activation which is associated with glycolysis [26, 27, 28] antibiotic‐induced microbiota disruption increased the phagocytic activity of intestinal macrophages (Figure 2j).

**FIGURE 3:**
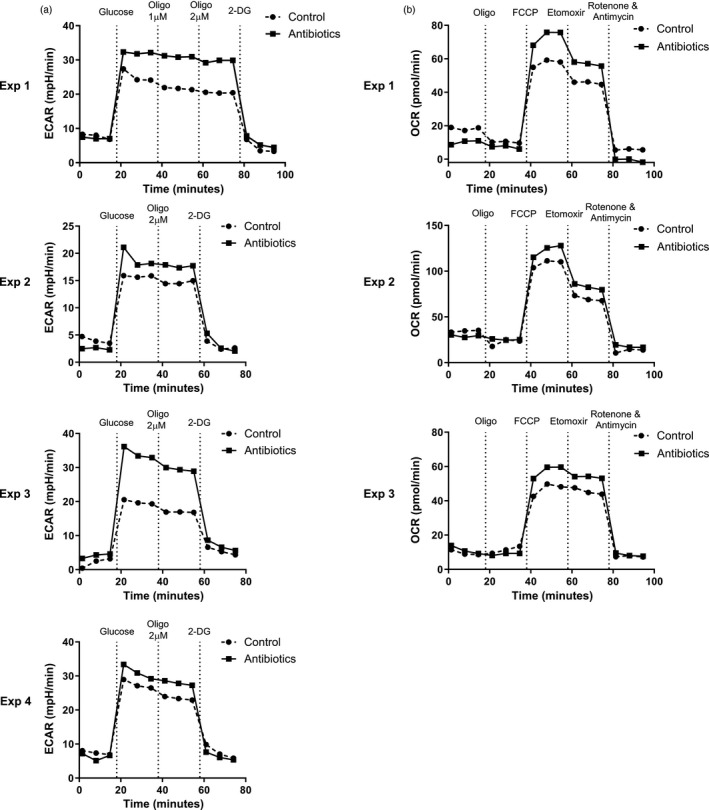
Seahorse analysis reveals increased metabolic output of large intestinal macrophages from antibiotic‐treated mice. Live CD45^+^SiglecF^−^Ly6G^−^CD11b^+^CD64^+^ large intestinal cells were sorted using flow cytometry from antibiotic‐ and control‐treated mice and analysed by Seahorse technology. (a) Extracellular acidification rate (ECAR) was measured after addition of 25 mm glucose, 1 µm and/or 2 µm oligomycin and 50 mm 2‐Deoxy‐d‐glucose (2‐DG). Four independent experiments are shown, in each case pooled sorted cells from five or six mice per group. (b) Oxygen consumption rate (OCR) was measured after addition of 2 µm oligomycin, 1·5 µm carbonyl cyanide‐4‐(trifluoromethoxy) phenylhydrazone (FCCP), 200 µm etomoxir and 100 nm rotenone and 1 µm antimycin A. Three independent experiments are shown, in each case pooled sorted cells from five or six mice per group

To confirm whether antibiotic‐induced microbial disruption shaped glycolysis and OXPHOS directly, we assessed the metabolic functions of sorted CD11b^+^ CD64^+^ colonic monocyte‐macrophages *ex vivo* using Seahorse^TM^ technology. Pooled cells from antibiotic‐treated mice exhibited increased ECAR as a measure of glycolysis across four independent experiments (Figure 3a), as well as increased OCR as a measure of OXPHOS [29] (Figure 3b) across three separate experiments. These data suggest colonic macrophages become increasingly metabolically active in response to antibiotics, demonstrating increased levels of glycolysis and OXPHOS. In terms of associating metabolic function with activation, these data are in keeping with our previous studies demonstrating antibiotic treatment induces pro‐inflammatory changes in intestinal macrophages [19].

### Macrophages from the small intestine are less metabolically active than their colonic counterparts with limited impact of antibiotics on their metabolic function

The gastrointestinal tract varies considerably in terms of function, environment and composition of the local microbiota, with a reduced microbial load and diversity in the small intestine [30, 31]. We assessed whether macrophages from the small intestine versus colon differ in their metabolic response to antibiotic treatment.

**FIGURE 4:**
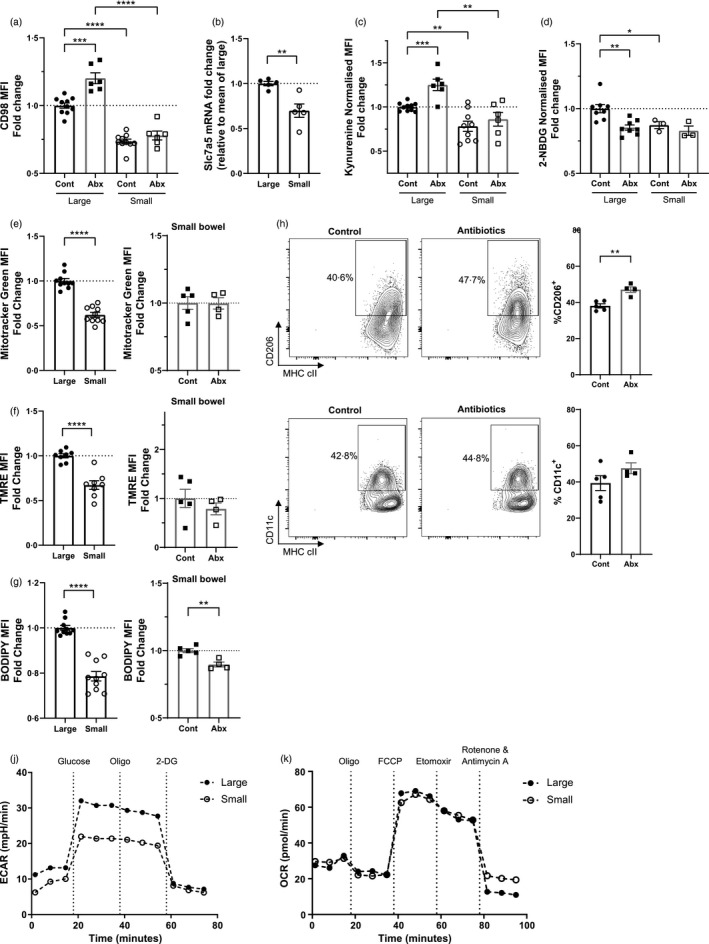
Small intestinal macrophages are less metabolically active, and less metabolically dependent on the gut microbiota. Mice were treated with antibiotic or control water and intestinal monocytes‐macrophages (live CD45^+^Ly6G^−^CD11b^+^CD64^+^) were identified from the large or small bowel by flow cytometry. (a) Mean fluorescent intensity (MFI) of CD98 protein expression on intestinal macrophages (Ly6C^−^MHC cII^+^). Fold change is normalized to mean of large control; *n* = 6–10. (b) Relative mRNA expression of Slc7a5 on sorted Ly6C^−^MHC cII^+^ macrophages. *n* = 5. (c) Intestinal cell suspensions were treated with kynurenine and cell uptake measured on Ly6C^−^MHC cII^+^ intestinal macrophages at 450 nm. MFI readings were normalized to background. Fold change is relative to the mean of the large control group; *n* = 6–10. (d–g) Intestinal cell suspensions were treated with (d) 2‐NBDG, (e) Mitotracker Green, (f) TMRE or (g) BODIPY FL C16 and cell uptake measured on Ly6C^−^MHC cII^+^ intestinal macrophages at 530 or 561 nm. MFI readings were normalized to background. Fold change is relative to the mean of the large control groups; *n* = 3–10. (h–i) CD206 (h) and CD11c (i) expression and on Ly6C^−^MHCcII^+^ macrophages by flow cytometry; *n* = 4–5. (j) Extracellular acidification rate (ECAR) was measured after addition of 25 mm glucose, 2 µm oligomycin and 50 mm 2‐Deoxy‐d‐glucose (2‐DG). (K) Oxygen consumption rate (OCR) was measured after addition of 2 µm oligomycin, 1·5 µm carbonyl cyanide‐4‐(trifluoromethoxy) phenylhydrazone (FCCP), 200 µm etomoxir and 100 nm rotenone and 1 µm antimycin A. *p < 0·05, **p < 0·01, ***p < 0·001, ****p < 0·0001, Student's *t*‐test

Small intestinal macrophages expressed lower levels of the amino acid transporter LAT1, with reduced CD98 protein (Figure 4a) and Slc7a5 mRNA (Figure 4b) compared to their colonic counterparts. The uptake of kynurenine (Figure 4c) and the florescent glucose mimic 2‐NBDG (Figure 4d) was also reduced on macrophages in the small intestine, indicating restricted LAT1‐mediated amino acid and glucose transport respectively. Furthermore, small bowel macrophages had a lower mitochondrial mass (Figure 4e), mitochondrial membrane potential (Figure 4f) and uptake of fatty acid (Figure 4g). Accordingly, antibiotic treatment had limited effects on metabolic functioning of macrophages from the small intestine, with LAT1‐mediated amino acid uptake, expression of CD98, glucose uptake, mitochondrial mass and respiration all remained unchanged in contrast to macrophages from the colon (Figure 4a,c–f), although fatty acid uptake was decreased (Figure 4g). Interestingly, in contrast to the colon (Figure 2h,i), the expression of CD11c by small intestinal macrophages was unaffected by antibiotic treatment while there was a modest increase in CD206 (Figure 4h,i). Finally, glycolysis and OXPHOS from CD11^+^ CD64^+^ monocyte‐macrophages were analysed by Seahorse. ECAR of macrophages from the small intestine was lower compared to those of the colon, indicating lower levels of glycolysis (Figure 4h), but OCR was unchanged (Figure 4i). Collectively, these data indicate that macrophages in the small intestine are less metabolically active than colonic macrophages, demonstrating reduced amino acid uptake, glycolysis, mitochondrial respiration and lipid metabolism, and that microbial‐induced disruption of the microbiota has limited effects on macrophages in the small intestine.

### Lipid uptake is a characteristic of intestinal tissue‐resident macrophage metabolism

**FIGURE 5:**
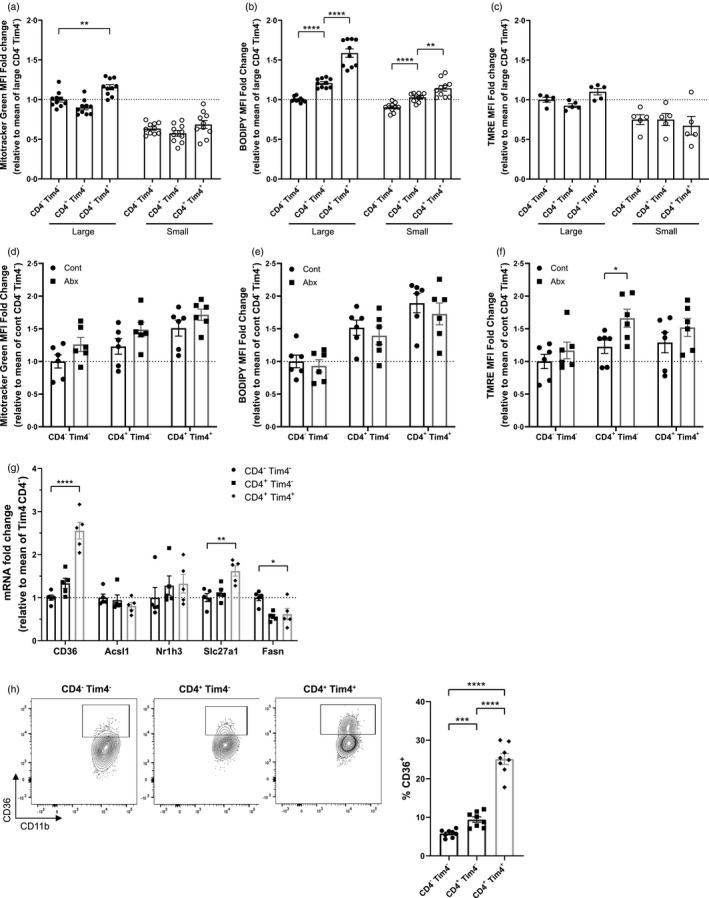
Fatty acid uptake defines the metabolism of intestinal tissue‐resident macrophages. Large and small bowel cell suspensions from naïve mice were isolated and Tim4^−^CD4^−^, Tim4^−^CD4^+^ and Tim4^+^CD4^+^ macrophages identified by flow cytometry. (a) Cells were treated with 50 nm Mitotracker Green and staining measured at 530 nm. *n *= 10. (b) Cells were treated with 25 nm of TMRE and fluorescence measured at 586 nm. *n* = 5. (c) Cells were treated with 5 µm BODIPY FL C16 in fatty acid‐free media, and uptake measured at 530 nm, *n* = 10. (d–f) Mice were treated with antibiotic‐treated or control drinking water, colonic macrophage subsets were stained as in (a–c) with Mitotracker Green (d), BODIPY FL C16 (e) and TMRE (f), *n* = 6. (g) Large intestinal macrophages (live CD45^+^SiglecF^−^Ly6G^−^CD11b^+^CD64^+^Ly6C^−^ MHCcII^+^) were sorted by flow cytometry into Tim4^−^CD4^−^, Tim4^−^CD4^+^ and Tim4^+^ CD4^+^ subsets, and mRNA expression of lipid uptake and synthesis‐related genes was measured. *N* = 5. (h) CD36 protein expression on large intestinal macrophages (Ly6C^−^MHCcII^+^) by flow cytometry; *n* = 8. *p < 0·05, **p < 0·01, ***p < 0·001, ****p < 0·0001, Student's *t*‐test

Given the limited effects of antibiotic‐induced microbial disruption on tissue‐resident subsets of intestinal macrophages with slow turnover from the circulation in terms of induction of pro‐inflammatory properties [19], we aimed to investigate whether subsets of intestinal macrophages exhibited differences in their metabolic capacities. Macrophage subsets were characterized as replenished (CD4^−^Tim4^−^ and CD4^+^Tim4^−^ macrophages) or tissue‐resident (CD4^+^Tim4^+^) in the colon. In the steady state, although mitochondrial mass (Figure 5a) and membrane potential (Figure 5b) were comparable between the CD4^−^Tim4^−^, CD4^+^Tim4^−^ and CD4^+^Tim4^+^ macrophages within the small intestine and colon separately, the uptake of lipids as measured by BODIPY C16 was significantly higher in CD4^+^Tim4^+^ double positive macrophages with the slowest turnover rate in both compartments (Figure 5c). To determine whether antibiotic treatment affects the metabolism of macrophage subsets differently, colonic macrophages were isolated from antibiotic‐treated mice and cellular metabolism assessed as above. Antibiotic treatment did not significantly alter the mitochondrial mass (Figure 5d) or fatty acid uptake (Figure 5e) in any macrophage subset. However mitochondrial potential was significantly elevated in the subset of CD4^+^Tim4^−^ macrophages (Figure 5f), suggesting that the increased TMRE staining in total gut macrophages from antibiotic‐treated mice (Figure 2e) may be due to metabolic changes in CD4^+^Tim4^−^ replenished macrophages.

To confirm whether a potential increased in fatty acid uptake by resident CD4^+^Tim4^+^ colonic macrophages compared to other subsets was reflected by changes in the expression in relevant genes, mRNA was isolated from sorted CD4^+^Tim4^+^ colonic macrophage subsets and lipid metabolism genes quantified. Accordingly, the expression of fatty acid uptake genes including the scavenger receptor *Cd36* and the primary macrophage fatty acid transporter *Slc27a1* increased in the CD4^+^Tim4^+^‐resident macrophages (Figure 5d). In contrast to this, *Fasn*, the primary fatty acid synthase, was downregulated in the double positive population (Figure 5d). The protein expression of CD36 was also significantly elevated in the CD4^+^Tim4^+^ subset (Figure 5e). These data suggest that the capacity for fatty acid uptake differs according to the subset of colonic macrophages. Fatty acid uptake was greatest in the tissue‐resident CD4^+^Tim4^+^ subset, which may be reflective of reduced *de novo* synthesis as indicated by the reduced expression of *Fasn*. This is a characteristic of other tissue‐resident immune cells [32], and likely has implications for differing functions of tissue‐resident versus recruited macrophages.

## DISCUSSION

We demonstrate that intestinal macrophages can constitutively take up amino acids from their local environment and that this is regulated by the intestinal microbiota. Antibiotic‐induced microbial disruption further enhanced LAT1‐mediated amino acid uptake and as such, changed the metabolic capacities of macrophages in the intestine. Taken with the direct association between cellular metabolism and activation [6, 25, 28, 33, 34], these data provide novel insight into pathways by which macrophage activation in the intestine may be regulated by the commensal microbiota to avoid inappropriate inflammation. The reduced metabolic capacity of macrophages in the small intestine is likely to be due to the environmental differences between the different regions of the intestine [30, 31] but has important implications for macrophage function. The small bowel is responsible for the absorption and digestions of protein, monosaccharides and vitamins; hence immune cell homeostasis is more dependent on diet in the small intestine compared to the colon [35]. Yet the restricted metabolic capacity of small intestinal macrophages compared to their colonic counterparts indicate that the intestinal microbiota may play a bigger role in shaping macrophage metabolism in the intestine than diet—despite the obvious implications for availability of amino acids from dietary protein in the small intestine.

Unlike antibiotic effects on colonic macrophages, antibiotics did not alter the mitochondrial potential of small intestinal macrophages but reduced fatty acid uptake, indicating compartment‐specific effects of antibiotics on intestinal macrophage metabolism. These differences may at least in part be due to compartmental differences in local availability of metabolites such as SCFAs and dietary‐derived amino acids that are diminished by antibiotic administration. Indeed, SCFAs that are found at high concentration in the colon limited mitochondrial function in colonic macrophages, which was increased with antibiotic administration. The enhanced expression of CD11c (associated with classical activation of macrophages) alongside reduced expression of alternative activation marker CD206 induced by antibiotics on colonic macrophages indicates programming of a pro‐inflammatory profile on macrophages in the colon but not in the small intestine. These data indicate the importance of considering different compartments of the intestine when assessing the immunomodulatory effects of local microbiota and metabolites. These data also have implications for compartment‐specific dysregulation of macrophage function that can occur in inflammatory bowel disease—a disease characterized by an inappropriate immune response to the commensal microbiota.

We have previously demonstrated that intestinal macrophages from antibiotic‐treated mice exhibit pro‐inflammatory properties, responding more potently to bacterial‐derived LPS and expressing enhanced levels of iNOS, aligning with classically activated macrophages [19]. Although in vitro classically activated macrophages rely on glycolysis for energy production to enable rapid responses to pathogens when needed [36], we show here that antibiotic‐induced microbial disruption increases both glycolysis and OXPHOS pathways together in primary gut macrophages. Recent publications have shown enhanced glycolysis and OXPHOS occurring at the same time in immune cells under diverse conditions, including human natural killer cell activation [37] and in CD14^+^ monocytes isolated from rheumatoid arthritis patients [38]. Tissue‐associated macrophages, often thought as M2‐like and dependent on mitochondrial respiration, are also reported to have high glycolytic activity [39]. Our data support a role for the microbiota in limiting macrophage metabolism in the intestine, in contrast to the regulation of macrophage metabolism at other mucosal sites. For example, in the lung the microbiota plays a limited role in alveolar macrophage metabolism, which is thought to be shaped by local glucose levels [40]. Traditionally, a characteristic of alternatively activated macrophages is their utilization of OXPHOS for efficient energy production where downstream products of the TCA cycle such as itaconate inhibit inflammation through suppression of IL‐6 and IL‐1b [41, 42]. However, despite upregulation of both metabolic pathways in the colon, antibiotic treatment induced lower levels of alternative activation marker CD206 alongside higher amounts of CD11c (a classical activation marker). Furthermore, macrophage activation was increased with antibiotics as indicated by phagocytic activity, again supporting the induction of a pro‐inflammatory phenotype in macrophages by antibiotics as seen in our previous studies.

Our observation that Tim4^+^CD4^+^ tissue‐resident intestinal macrophages upregulate fatty acid transporters and scavengers while downregulating fatty acid synthesis genes raises the possibility that this subset utilizes the uptake of local fatty acids for function. CD36 has been defined as a key part of metabolic reprograming that occurs when CD8 memory T cells become resident in the skin [32, 43], suggesting CD36 may be involved in tissue residency. The uptake of lipids and their metabolism through beta‐oxidation in mitochondria grants these cells longevity in skin tissue. The differences in metabolism between macrophage subsets in the intestine are likely to impact on their function; as yet it is unclear what the practical differences between these recruited versus resident macrophages in the intestine are. Given CD36 expression is correlated with an immunosuppressive phenotype seen in tumour‐associated macrophages [44], taken with fatty acid uptake being a hallmark feature of alternatively activated macrophages [45], our data suggest that the functions of CD4^+^Tim4^+^ tissue‐resident macrophages may be more directed towards tissue remodelling and integrity rather than response to microbial stimulation. Indeed, Tim4^+^CD4^+^ macrophages produce lower levels of pro‐inflammatory type 1 cytokine IL‐6 compared to their Tim4^−^CD4^−^ recruited counterparts,^4^ whereas we show here that microbial modulation of intestinal macrophage function is most relevant for recruited CD4^+^Tim4^−^ macrophages. Further work is needed to elucidate the roles of specific macrophage subsets in intestinal immune homeostasis.

## CONFLICT OF INTEREST

The authors have no conflict of interest to declare.

## AUTHOR CONTRIBUTIONS

Nicholas A. Scott and Elizabeth R. Mann wrote the manuscript and designed the study. Nicholas A. Scott, Melissa A. E. Lawson, Lindsay J. Hall, Elizabeth R. Mann, Ryan James Hodgetts and Gwenaelle La Gall performed the experiments and analysed the data.

## Supporting information

Fig S1

Fig S2

Table S1‐S2
