# Supplementary material for: Macrophage metabolism in the intestine is compartment specific and regulated by the microbiota
Source: Immunology. 2022 Mar 11;166(1):138–52. doi: 10.1111/imm.13461 (PMC10357482; doi:10.1111/imm.13461)

SUPP FIGURE 2: Effect of amino acid and short chain fatty acid supplementation on intestinal macrophage metabolism

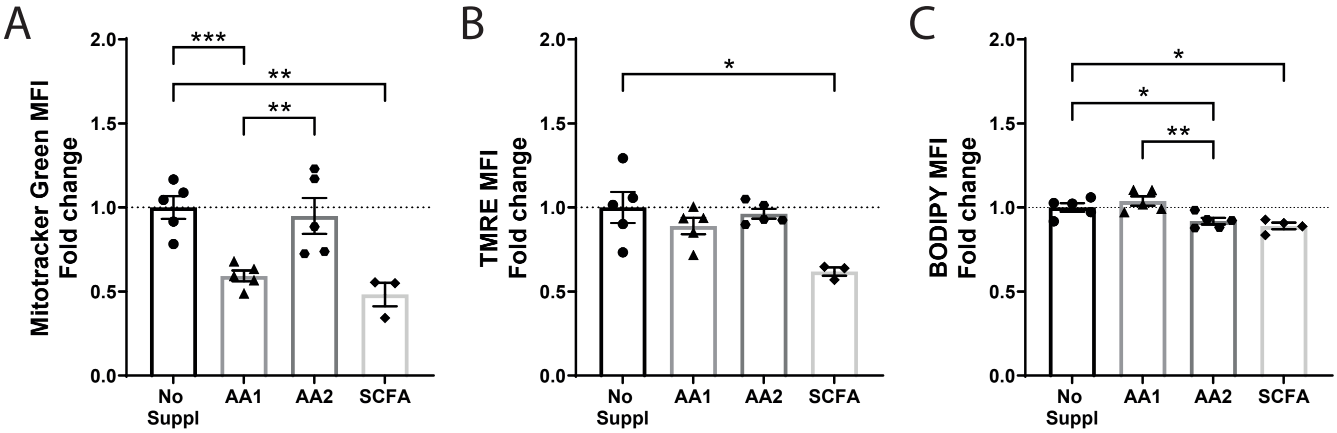

Supplement: Supplementary file 2 — Fig S2 [file IMM-166-138-s002.pdf]
