# Supplementary material for: Macrophage metabolism in the intestine is compartment specific and regulated by the microbiota
Source: Immunology. 2022 Mar 11;166(1):138–52. doi: 10.1111/imm.13461 (PMC10357482; doi:10.1111/imm.13461)
Supplement: Supplementary file 3 — Table S1‐S2 [file IMM-166-138-s003.docx]

**SUPPLEMENTARY TABLES**

**Table S1. Antibodies.**

| **Antibody** | **Conjugate** | **Clone** | **Source** | **Catalogue number** |
| --- | --- | --- | --- | --- |
| CD45 | FITC | 30-F11 | Biolegend | 103108 |
| CD45 | BV510 | 30-F11 | Biolegend | 103137 |
| CD11b | BV605 | M1/70 | Biolegend | 101257 |
| CD206 | BV421 | C068C2 | Biolegend | 141717 |
| CD4 | PE-Cy7 | RM4-5 | Biolegend | 100528 |
| CD64 | PE | X54-5/7.1 | Biolegend | 139304 |
| CD98 | 647 | RL388 | Biolegend | 128212 |
| CD11c | BV395 | HL3 | BD Horizon | 564080 |
| Ly6C | PerCP-Cy5.5 | HK1.4 | Biolegend | 128012 |
| Ly6G | BV510 | 1A8 | Biolegend | 127608 |
| MHC class II (I-A/I-E) | AF700 | M5/114.15.2 | Biolegend | 128024 |
| p-mTOR (S2448) | PE-Cy7 | MRRBY | Invitrogen | 25-9718-42 |
| p-Akt (S473) | e450 | SDRNR | eBioscience | 48-9715-42 |
| p-S6 (S235/S236) | APC | cupk43k | Invitrogen | 17-9007-42 |
| Siglec F | BV421 | E50-2440 | BD Biosciences | 562681 |
| Tim4 | APC | RMT4-54 | Biolegend | 130008 |

**Table S2. Primers sequences for gene expression.**

| Gene | 5’ | 3’ |
| --- | --- | --- |
| TATA BP | CTCAGTTACAGGTGGCAGCA | GCCCAAGTAGCAGCACAGA |
| Glut1 | ATACTCATGACCATCGCGCTAG | AAAGAAGGCCACAAAGCCAAAG |
| Ldha | TGGCAGACTTGGCTGACAG | ACCTTCACAACATCCGAGATTC |
| HIF-1a | GAAACGACCACTGCTAAGGCA | GGCAGACAGCTTAAGGCTCCT |
| Pdk1 | GCAGCAGAGAGTAAACTGTTTG | TGGTCACCTGACCTCTCG |
| HK2 | TGATCGCCTGCTTATTCACGG | AACCGCCTAGAAATCTCCAGA |
| PPARa | TCGGCGAACTATTCGGCTG | GCACTTGTGAAAACGGCAGT |
| PPARy | TGTGGGGATAAAGCATCAGGC | CCGGCAGTTAAGATCACACCTAT |
| LCAD | AACACCATGTATGCTCGGCTC | CCGACCAAGATAATCCCCTTGAG |
| UCP2 | CAGATGTGGTAAAGGTCCGCT | TTCCTCTCGTGCAATGGTCTT |
| ACADL | TCTTTTCCTCGGAGCATGACA | GACCTCTCTACTCACTTCTCCAG |
| cd36 | TCCAGCCAATGCCTTTGC | TGGAGATTACTTTTCAGTGCAGAA |
| acsl1 | TGCCAGAGCTGATTGACATTC | GGCATACCAGAAGGTGGTGAG |
| Nr1h3 | CTGATTCTGCAACGGAGTTGT | GACGAAGCTCTGTCGGCTC |
| Slc27a1 | TCTGTTCTGATTCGTGTTCGG | CAGCATATACCACTACTGGCG |
| Fasn | GGAGGTGGTGATAGCCGGTAT | TGGGTAATCCATAGAGCCCAG |
